# Ultrasound-Guided Pulsed Radiofrequency for Carpal Tunnel Syndrome: A Single-Blinded Randomized Controlled Study

**DOI:** 10.1371/journal.pone.0129918

**Published:** 2015-06-12

**Authors:** Liang-Cheng Chen, Cheng-Wen Ho, Chia-Hung Sun, Jiunn-Tay Lee, Tsung-Ying Li, Feng-Mei Shih, Yung-Tsan Wu

**Affiliations:** 1 Department of Physical Medicine and Rehabilitation, Tri-Service General Hospital, School of Medicine, National Defense Medical Center, Taipei, Taiwan, Republic of China; 2 Department of Physical Medicine and Rehabilitation, Hualien Armed Forces General Hospital, Hualien County, Taiwan, Republic of China; 3 Graduate Institute of Aerospace Medicine, School of Medicine, National Defense Medical Center, Taipei, Taiwan, Republic of China; 4 Department of Neurology, Tri-Service General Hospital, School of Medicine, National Defense Medical Center, Taipei, Taiwan, Republic of China; The James Cook University Hospital, UNITED KINGDOM

## Abstract

**Objective:**

We assessed the therapeutic efficiency of ultrasound-guided pulsed radiofrequency (PRF) treatment of the median nerve in patients with carpal tunnel syndrome (CTS).

**Methods:**

We conducted a prospective, randomized, controlled, single-blinded study. Forty-four patients with CTS were randomized into intervention or control groups. Patients in the intervention group were treated with PRF and night splint, and the control group was prescribed night splint alone. Primary outcome was the onset time of significant pain relief assessed using the visual analog scale (VAS), and secondary outcomes included evaluation of the Boston Carpal Tunnel Syndrome Questionnaire (BCTQ) results, cross-sectional area (CSA) of the median nerve, sensory nerve conduction velocity (SNCV) of the median nerve, and finger pinch strength. All outcome measurements were performed at 1, 4, 8, and 12 weeks after treatment.

**Results:**

Thirty-six patients completed the study. The onset time of pain relief in the intervention group was significantly shorter (median onset time of 2 days vs. 14 days; hazard ratio = 7.37; 95% CI, 3.04–17.87) compared to the control group (p < 0.001). Significant improvement in VAS and BCTQ scores (p < 0.05) was detected in the intervention group at all follow-up periods compared to the controls (except for the severity subscale of BCTQ at week 1). Ultrasound-guided PRF treatment resulted in a lower VAS score and stronger finger pinch compared to the control group over the entire study.

**Conclusions:**

Our study shows that ultrasound-guided PRF serves as a better approach for pain relief in patients with CTS.

**Trial Registration:**

ClinicalTrials.gov NCT02217293

## Introduction

Carpal tunnel syndrome (CTS) is the most common peripheral nerve entrapment neuropathy, which is caused by the compression of median nerve in the carpal tunnel. The average prevalence of CTS is 3–4% with a female predominance (7% female and 1% male), and it is more common in certain occupational populations such as computer users, meatpackers, and cashiers [1]. The symptoms of CTS are numbness, tingling, pain, or burning sensation of at least 2 of 3 digits innervated by the median nerve; thenar muscle atrophy might occur at later stages [2].

The diagnosis of CTS is based on clinical signs (Phalen’s test or Tinel’s sign) and is established using electrophysiological testing. Repetitive stress of the wrist, obesity, and pregnancy are the main risk factors for CTS, and secondary causes, such as lesions within the carpal tunnel, metabolic causes, and infection have been reported [2]. Unlike other progressive diseases, CTS is characterized by remissions and recurrences, and therefore, prognosis is often uncertain. Although many conservative treatment methods are applied; including the use of wrist splint, steroid injections, and therapeutic ultrasound; their effectiveness is typically insignificant or short-lived [3]. Indeed, Katz et al. [4] revealed that around 60–70% of patients with CTS treated conservatively remained symptomatic after 18 months. For wrist splinting, a treatment failure rate of 69% (57 of 83 patients) was reported after a 12-months follow-up period [5].

Pulsed radiofrequency (PRF) treatment is a relatively novel pain-intervention technique. It alleviates pain by delivering an electric impulses and heat bursts at a temperature of less than 42°C to avoid causing neuronal injury, contrary to conventional RF applications that apply a constant high temperature of 60° to 80°C [6]. PRF has been reported relieve pain in certain chronic pain conditions [6–8]; however, the application of PRF in CTS is scarce. Haider et al. [9] described a patient with recurrent CTS, who experienced a significant 70% reduction of symptoms over 12 weeks after ultrasound-guided (UG) PRF treatment.

The purpose of this study was to assess the analgesic effect of ultrasound-guided PRF in prognosis of the median nerve in patients with CTS.

## Methods

The protocol for this trial and supporting CONSORT checklist are available as supporting information; see S1 CONSORT Checklist and S1 Protocol.

### Ethics Statement

This study was reviewed and ethically approved by the Institutional Review Board (IRB) and Ethics Committee of the Tri-Service General Hospital, National Defense Medical Center, Taiwan (No. 1-101-05-049). The study was performed according to the principles of Declaration of Helsinki. Subjects gave their written, informed consent for the study. This study was registered retrospectively at clinicaltrials.gov (NCT02217293) due to administrative difficulties with the registration process. Authors confirm that all ongoing and related trials for this intervention are registered. No important changes have been made in the methods after trial commencement.

### Study Design

This was a prospective, randomized, controlled, single-blinded study conducted in the Tri-Service General Hospital, Taiwan. From November 1, 2012 to September 15, 2013, 80 patients diagnosed with CTS were assessed for eligibility, and 44 were enrolled in the study.

**Fig 1:**
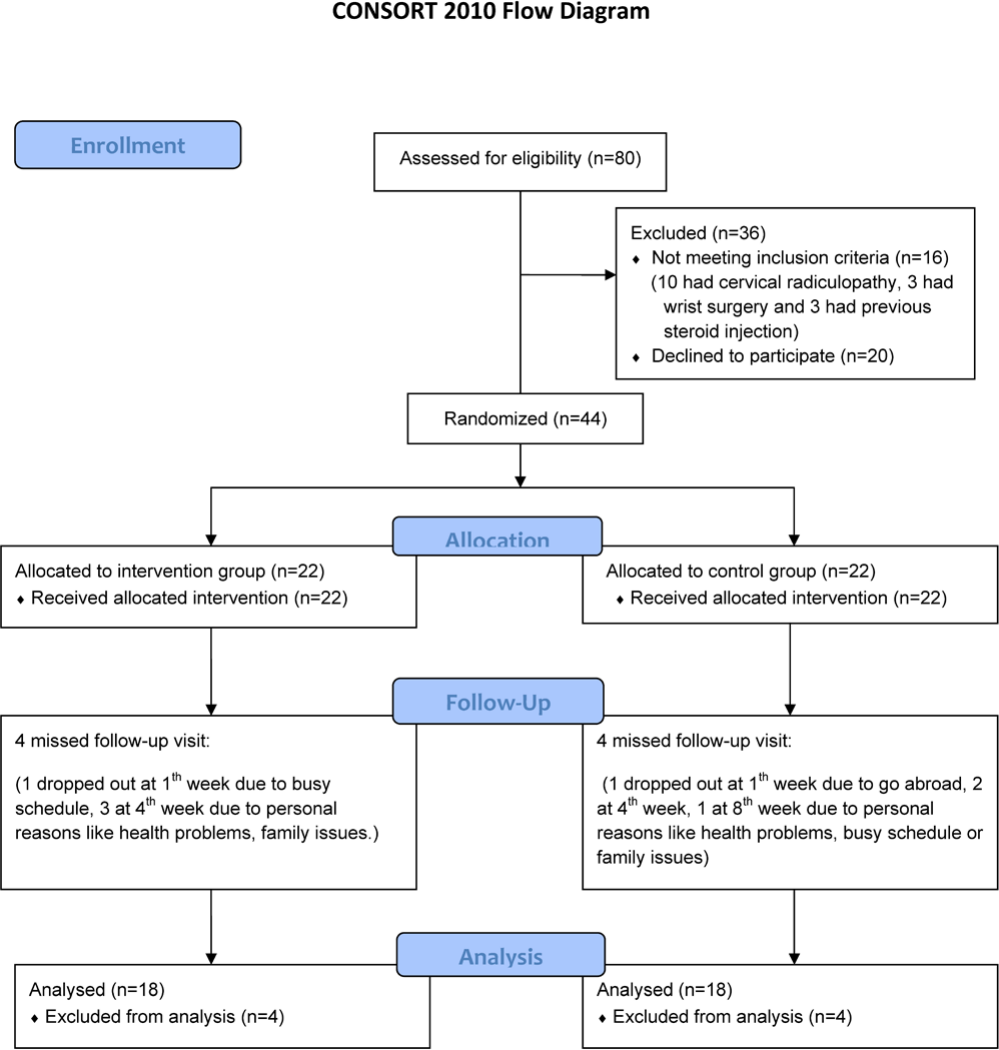
Study flow diagram.

Patients were block randomized with a 1:1 ratio (block size = 4, allocation using a fixed block size of 4 to divide into 11 blocks from 44 participants, one block could determine two intervention groups and two control groups) by a research assistant who drew numbers from a sealed envelope, a number in the sealed envelope which created from a random-number table to avoid the participants to know their subgroups (intervention or control), into the intervention or control groups (see Fig 1 for the flow diagram of enrollment). In the intervention group, participants received one dose of PRF, and the control group did not receive PRF treatment.

To provide fundamental medical care for CTS, night wrist splint was prescribed for each subject in both groups. Night wrist splint was firmly fixed in a neutral position to immobilize the affected wrist [3]. Patients were ordered to wear the splint while resting at night and at least 8 hours a day during the period of study (12 weeks). Patients were instructed to keep away from any other treatment for their pain or discomfort resulting from CTS including analgesic agents, injections, or acupuncture etc. from initial screening throughout the study period. They were asked to notify us if they had taken any of these therapies.

### Inclusion and Exclusion Criteria

Patients were enrolled in the study if they had typical symptoms and signs of CTS, such as positive Tinel’s sign, Phalen’s test, numbness, or tingling in at least two digits of the hand, and were confirmed with CTS by electrophysiological tests. Patients who had conditions mimicking CTS, such as cervical radiculopathy, polyneuropathy, brachial plexopathy, thoracic outlet syndrome, had previous wrist surgery, or received steroid injections for CTS, were all excluded.

### Ultrasound-Guided PRF for the Median Nerve

**Fig 2:**
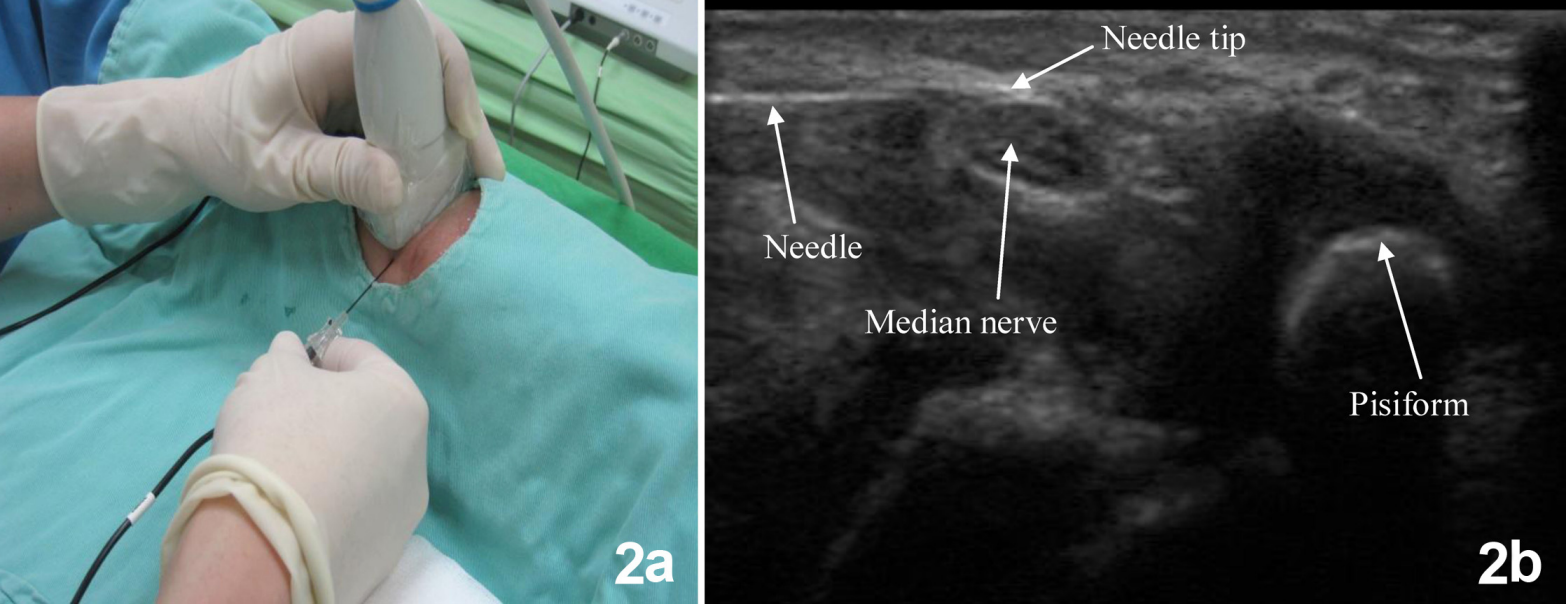
a: Positioning of the ultrasonographic transducer and radiofrequency needle. b: Real-time imaging of the ultrasound-guided needle insertion.

Musculoskeletal ultrasonography (Terason, t3000, USA) was performed by the same physician (Dr. Chen). Patients were seated in a relaxed position with their forearm and fingers resting on a table with their palm facing upwards. The median nerve was identified at the proximal carpal tunnel (pisiform level) (Fig 2A) [10]. Then a 54-mm radiofrequency probe with a 4-mm active tip (Neurotherm NT1000, Neurotherm Inc., USA) was advanced with ultrasound guidance using the in-plane technique towards the median nerve (Fig 2B). Sensory and motor stimulation was tested when the needle was close to the median nerve. During sensory stimulation (50–100 Hz; 1-ms pulsed width; up to 0.5 volt), the patients reported paresthesia in the distal fingers. After performing a motor stimulation (2 Hz; 1-ms pulsed width; up to 1 volt), contractions of the thenar muscle were observed. PRF lesion was then carried out for 120 seconds at a 2 Hz frequency and pulse width of 20 ms at 42°C. Thirty minutes after the PRF lesion, pain was evaluated in all patients by the same physician (Dr. Sun) using the visual analog scale (VAS). Patients were discharged with no significant complications such as pain, bleeding, or ongoing paresthesia.

### Outcome Measurements

All the measurements were performed by the same physician (Dr. Sun), who was blinded to the randomization and treatment procedure. Evaluation was performed before intervention and 1, 4, 8, and 12 weeks after the treatment.

### Primary Outcome

#### Onset time of significant pain relief

The VAS was used to quantify pain on the scale of 0 (no pain) to 10 (extremely severe pain) [11]. The severity of pain was scaled for any activity done regularly during the day. Each patient recorded the VAS score every day at the same time, until they reached the onset time of significant pain relief for 2 consecutive days. Onset time was defined as the day when the VAS score declined by 40% or more [12, 13].

### Secondary Outcomes

#### a. Boston Carpal Tunnel Syndrome Questionnaire (BCTQ) (See S1 Appendix)

BCTQ is the most commonly used questionnaire in clinical studies for evaluation of the symptom severity and functional status of patients with CTS [14]. Symptom severity is rated based on 11 questions scored from 1 point (mildest) to 5 points (most severe), and the functional status is evaluated with 8 questions scored from 1 point (no difficulty with activity) to 5 points (cannot perform the activity at all).

#### b. Cross-sectional area (CSA)

CSA of the median nerve was measured at the proximal inlet of the carpal tunnel (at the level of the pisiform bone) by the same physician [10]. The patients held their wrists in a neutral position with the palm up and the fingers semi-extended. CSA was measured three times, and the mean was used for the analysis. The ultrasonographic evaluation of median nerve CSA has high sensitivity (89%) and specificity (83%) for the diagnosis of CTS [10].

#### c. Sensory nerve conduction velocity (SNCV)

The antidromic SNCV of the median nerve was measured in all subjects according to the protocol reported by the American Academy of Neurology using SierraWave (Cadwell, USA) [15]. All examinations were performed by the same physician in the same room maintained at a constant temperature of 25°C. Skin temperature on the hand and wrist was maintained between 32.0 and 34.0°C. Active and reference ring electrodes were placed over the 2^nd^ proximal and distal interphalangeal joints. The median nerve was stimulated at the wrist between the palmar longus and flexor carpal radialis tendon at a distance of approximately 14 cm from the active electrode.

#### d. Finger pinch

Finger pinch strength was measured using the Jamar dynamometer (Fabrication Enterprises Inc., USA). The subject was seated, and the shoulder was neutrally rotated with the elbow flexed at 90°. The forearm and wrist were positioned in a neutral position for the palmar pinch [16]. The finger pinch was tested three times, and the mean was used for analysis.

### Sample Size

There are no published studies investigating ultrasound-guided PRF for CTS, and our research is a preliminary and novel study in this field. Therefore, no previous study could be referenced before designing the experiment and deciding the number of patients.

### Data Analysis

Statistical analyses were performed using the IBM SPSS Statistics Version 22 (Asia Analytics Taiwan Ltd., Taipei, Taiwan). Demographic data were described by means, standard deviations, and percentages. Outcomes at each follow-up period were compared with baseline values, and differences between groups were investigated using the paired t-test and χ^2^ test. We used the Kaplan-Meier analysis and the log-rank test to compare the rate of significant pain relief between the groups (event was defined as > 40% pain relief). In addition, Cox regression analysis was used to estimate hazard ratios (HRs) and 95% confidence intervals (CIs) for pain relief between intervention and control groups. All statistical tests were two-tailed, and statistical significance was set at p < 0.05.

## Results

Thirty-six patients completed the study, and both groups equally consisted of 18 cases. The reason of dropout was due to patients’ personal reason including health problem, family issues or busy schedule (see Fig 1). Baseline demographic data and clinical characteristics of the study population are summarized in Table 1; there was no significant difference between the groups regarding these factors (p > 0.05). Table 2 presents the VAS scores, BCTQ scores, finger pinch strength, CSA, and SNCV of the median nerve before and after treatment. Significant improvement of VAS and BCTQ scores was detected in the intervention group at all observed times (except in the case of severity subscale of BCTQ at week 1) compared to the control group (p < 0.05).

**Table 1:** Baseline demographic and clinical characteristics of study participants.

	Intervention group (n = 18)	Control group (n = 18)
Body height (cm) (SD)	155.6 ± 5.6	155.4 ± 6.6
Diabetes mellitus (n)	3	3
Hypertension (n)	2	4
Body weight (kg) (SD)	61.5 ± 11.7	61.1 ± 8.4
Age (year) (SD)	54.8 ± 4.4	57.3 ± 5
Duration (months) (SD)	20.8 ± 13.4	22.9 ± 13.4
Dominant hand (right) (n)	18	18
Female/Male (n)	18/0	17/1
Lesion site		
Right side	10	12
Left side	8	6
VAS	5.4 ± 2.1	5 ± 1.5
BCTQ-severity	33.4 ± 6.4	33 ± 6.5
BCTQ-function	23.3 ± 3.4	23.2 ± 6.2
FP (kg)	3.4 ± 1.4	3.1 ± 1.5
SNCV (m/s)	30.1 ± 7.6	29.9 ± 8.1
CSA (mm^2^)	14.7 ± 1.8	13.8 ± 2.1

VAS = Visual analog scale; BCTQ = Boston Carpal Tunnel Syndrome Questionnaire; FP = Finger pinch; SNCV = Sensory nerve conduction velocity; CSA = Cross-sectional area.

**Table 2:** All the measured parameters before and after treatment.

	Intervention group (n = 18)			Control group (n = 18)			*p* value
	Mean ± SD	Mean difference (95% CI)	Difference (Mean ± SD) (%)	Mean ± SD	Mean difference (95% CI)	Difference (Mean ± SD) (%)	
VAS-Pre	5.4 ± 2.1			5.0 ± 1.5			0.552
VAS Wk1	2.5 ± 1.4	-2.9 ± 1.1 (2.4–3.4)	-55.7 ± 14.9	3.9 ± 1.1	-1.1 ± 1.2 (0.5–1.7)	-20.8 ± 19.1	0.002
VAS Wk4	2.2 ± 1.5	-3.2 ± 1.1 (2.6–3.7)	-61.6 ± 18.5	3.4 ± 1.3	-1.6 ± 1.4 (0.9–2.2)	-29.8 ± 25.4	0.013
VAS Wk8	1.8 ± 1.2	-3.6 ± 1.6 (2.8–4.4)	-67.5 ± 18.9	3.2 ± 1.1	-1.8 ± 1.5 (1.0–2.5)	-32.6 ± 27.6	<0.001
VAS Wk12	1.1 ± 0.8	-4.2 ± 2.1 (3.2–5.2)	-77.2 ± 15.1	3.0 ± 1.2	-2.0 ± 1.8 (1.1–2.9)	-36.7 ± 32.5	<0.001
BCTQs-Pre	33.4 ± 6.4			33.0 ± 6.5			0.837
BCTQs Wk1	21.7 ± 7.3	-11.7 ± 4.7 (9.4–14.1)	-35.6 ± 12.8	26.0 ± 6.7	-7.0 ± 6.6 (3.7–10.3)	-20.3 ± 18.7	0.077
BCTQs Wk4	19.6 ± 4.8	-13.9 ± 5.1 (11.3–16.4)	-41.1 ± 11.9	23.6 ± 6.2	-9.4 ± 7.7 (5.6–13.3)	-26.9 ± 21.2	0.037
BCTQs Wk8	17.1 ± 3.6	-16.3 ± 6.3 (13.2–19.4)	-47.9 ± 11.9	22.8 ± 6.7	-10.2 ± 8.7 (5.9–14.5)	-28.9 ± 25.3	0.004
BCTQs Wk12	13.7 ± 2.1	-19.7 ± 6.7 (16.4–23.0)	-57.8 ± 9.1	22.1 ± 6.3	-10.9 ± 9.2 (6.3–15.5)	-30.6 ± 25.2	<0.001
BCTQf-Pre	23.3 ± 3.4			23.2 ± 6.2			0.948
BCTQf Wk1	12.1 ± 3.9	-11.2 ± 4.6 (8.9–13.4)	-47.5 ± 17.2	16.3 ± 6.9	-6.9 ± 6.3 (3.8–10)	-28.7 ± 23.5	0.032
BCTQf Wk4	11.2 ± 3.8	-12.1 ± 4.3(10–14.3)	-51.7 ± 16.3	14.6 ± 4.9	-8.6 ± 6.8 (5.2–11.9)	-34.5 ± 22.1	0.025
BCTQf Wk8	10.7 ± 3.7	-12.6 ± 5(10.1–15.1)	-53.4 ± 17.2	14.4 ± 5.0	-8.8 ± 7.0 (5.3–12.2)	-35.4 ± 23.8	0.016
BCTQf Wk12	9.1 ± 2.2	-14.2 ± 4.1(12.2–16.3)	-60.4 ± 11.4	13.9 ± 5.0	-9.3 ± 7.4 (5.6–13.0)	-37.2 ± 25.4	0.001
FP-Pre (kg)	3.4 ± 1.4			3.1 ± 1.5			0.526
FP Wk1	4.5 ± 1.6	1.0 ± 0.6 (-1.3–-0.7)	35.1 ± 22.2	3.8 ± 1.8	0.7 ± 0.6 (-1.0–-0.4)	24.3 ± 18.8	0.282
FP Wk4	5.0 ± 1.3	1.6 ± 0.8 (-2.0–-1.2)	61.6 ± 53.5	4.2 ± 1.8	1.1 ± 1.2 (-1.7–-0.5)	42.7 ± 42.6	0.169
FP Wk8	5.4 ± 1.0	1.9 ± 0.9 (-2.4–-1.5)	82.6 ± 83.7	4.8 ± 1.7	1.6 ± 1.0 (-2.2–-1.1)	63.9 ± 43.4	0.205
FP Wk12	5.7 ± 1.1	2.3 ± 1.0 (-2.8–-1.8)	94.5 ± 90.2	4.9 ± 1.8	1.8 ± 1.2 (-2.4–-1.2)	69.5 ± 48.5	0.138
SNCV-Pre (m/s)	30.1 ± 7.6			29.9 ± 8.1			0.929
SNCV Wk1	30.3 ± 7.5	0.1 ± 2.2 (-1.3–1.0)	0.8 ± 8.7	30.2 ± 8.2	0.3 ± 1.0 (-0.8–0.2)	1.0 ± 3.3	0.970
SNCV Wk4	30.8 ± 7.5	0.6 ± 1.4 (-1.3–0.1)	2.4 ± 5.9	30.1 ± 8.2	0.2 ± 1.1 (-0.7–0.3)	0.7 ± 3.7	0.803
SNCV Wk8	30.3 ± 7.4	0.2 ± 2.0 (-1.2–0.8)	1.2 ± 7.7	30.3 ± 8.4	0.4 ± 1.2 (-1.0–0.2)	1.3 ± 4.6	0.987
SNCV Wk12	30.7 ± 7.6	0.5 ± 1.6 (-1.3–0.3)	2.1 ± 6.0	30.0 ± 8.2	0.1 ± 2.4 (-1.3–1.1)	0.7 ± 8.1	0.800
CSA-Pre (mm^2^)	14.7 ± 1.8			13.8 ± 2.1			0.221
CSA Wk1	13.0 ± 2.2	-1.7 ± 1.3 (1.0–2.3)	-11.5 ± 8.5	13.1 ± 2.2	-0.8 ± 0.5 (0.5–1.1)	-5.7 ± 4.0	0.940
CSA Wk4	12.3 ± 2.4	-2.4 ± 1.6 (1.6–3.2)	-16.5 ± 10.8	12.2 ± 2.6	-1.6 ± 1.0 (1.1–2.1)	-12.1 ± 7.3	0.947
CSA Wk8	11.5 ± 2.3	-3.2 ± 1.6 (2.4–4.0)	-21.8 ± 10.3	12.1 ± 2.6	-1.7 ± 1.4 (1.0–2.4)	-12.7 ± 9.8	0.455
CSA Wk12	11.0 ± 2.2	-3.7 ± 1.5 (2.9–4.4)	-25.3 ± 9.8	11.9 ± 2.2	-1.9 ± 1.4 (1.2–2.7)	-13.8 ± 10.2	0.233

Pre = Pretreatment; CI = Confidence intervals; VAS = Visual analog scale; BCTQ = Boston Carpal Tunnel Syndrome Questionnaire (s = severity and f = function); FP = Finger pinch; SNCV = Sensory nerve conduction velocity; CSA = Cross-sectional area; WK = Week.

**Fig 3:**
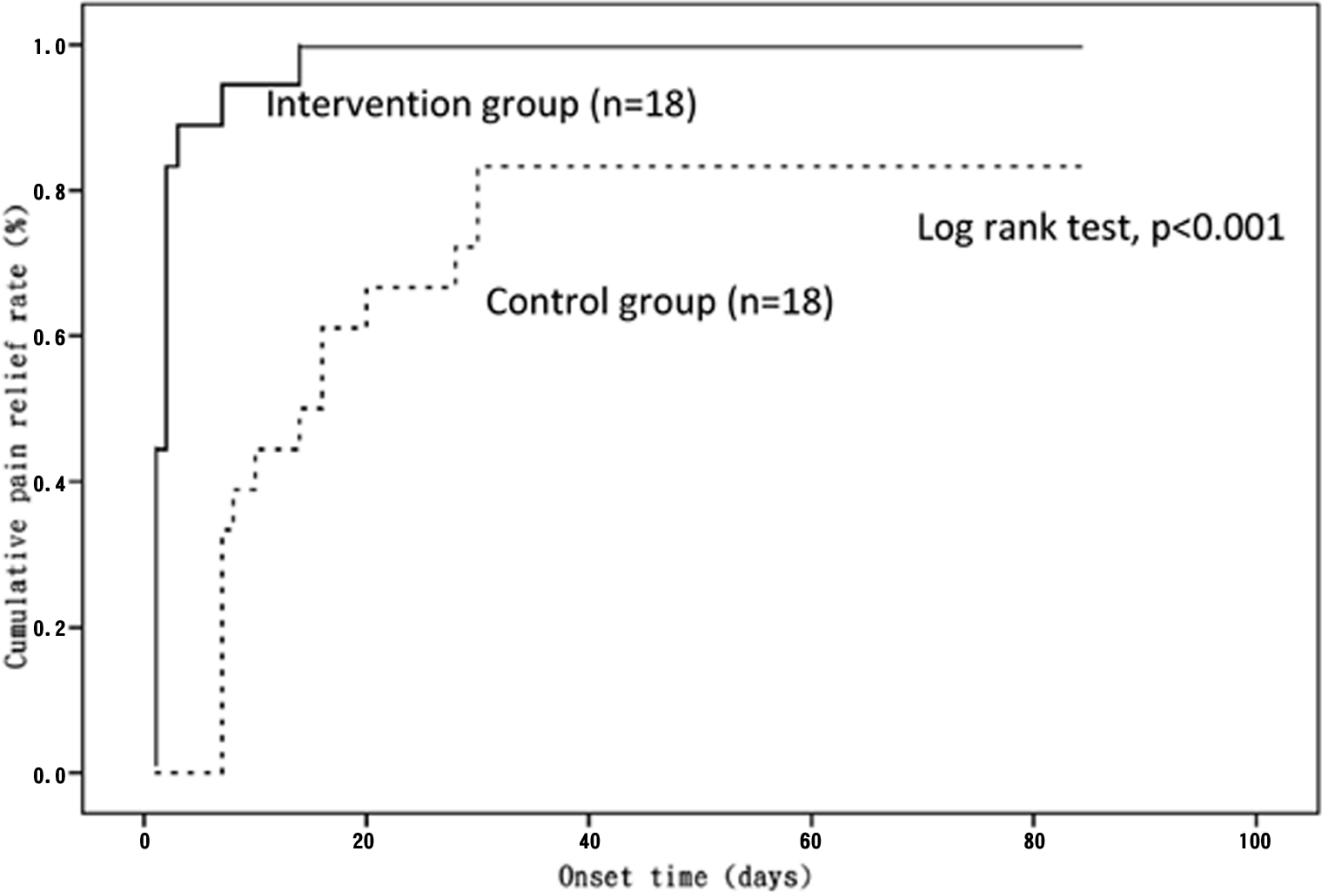
Kaplan-Meier survival analysis of pain relief rate.

Median onset time of significant pain relief was 2 days in the intervention group versus 14 days in the control group (p < 0.001). Hazard ratio for the onset time of significant pain relief in the intervention group versus control group was 7.37 (95% CI, 3.04 to 17.87, p < 0.001) (Table 3). Log-rank test revealed that the intervention group had significantly higher pain relief rate than the control group (p < 0.001). The Kaplan-Meier curves show significant pain relief rate in both groups (Fig 3).

**Table 3:** Intervention and control group of Kaplan-Meier and Cox regression analysis.

	Kaplan-Meier survival analysis			Cox regression analysis		
Group	No. of patients	Median onset time (days)	P value	HR	95% CI	P value
Control	18	14	<0.001	1.00	Reference	
Intervention	18	2		7.37	3.04–17.87	<0.001

HR = Hazard Ratio; CI = Confidence Interval

**Fig 4:**
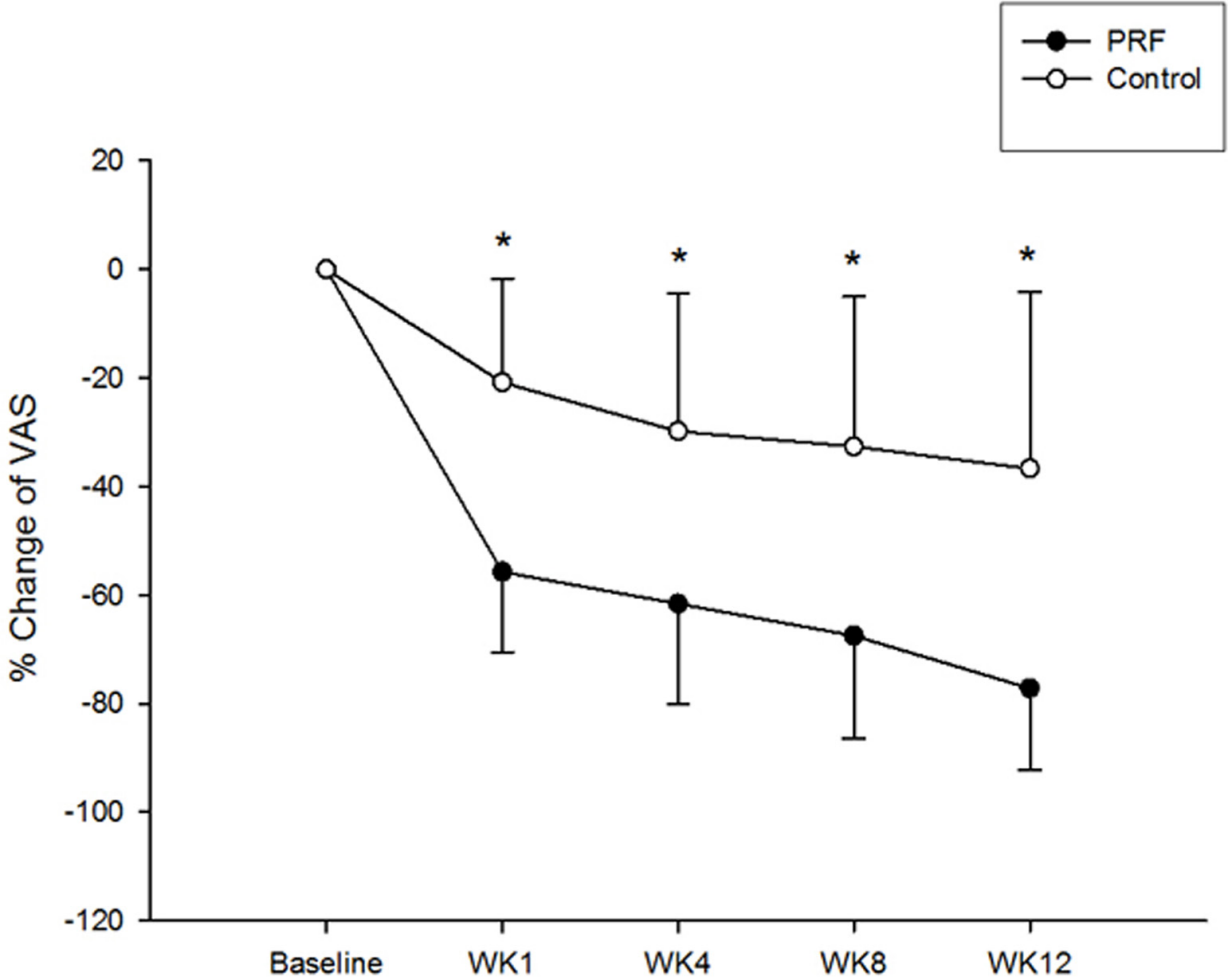
The percentage change in visual analog scale (VAS) scores (mean ± standard deviation) in both groups at different times. *Denotes significant difference between groups, p < 0.001.

**Fig 5:**
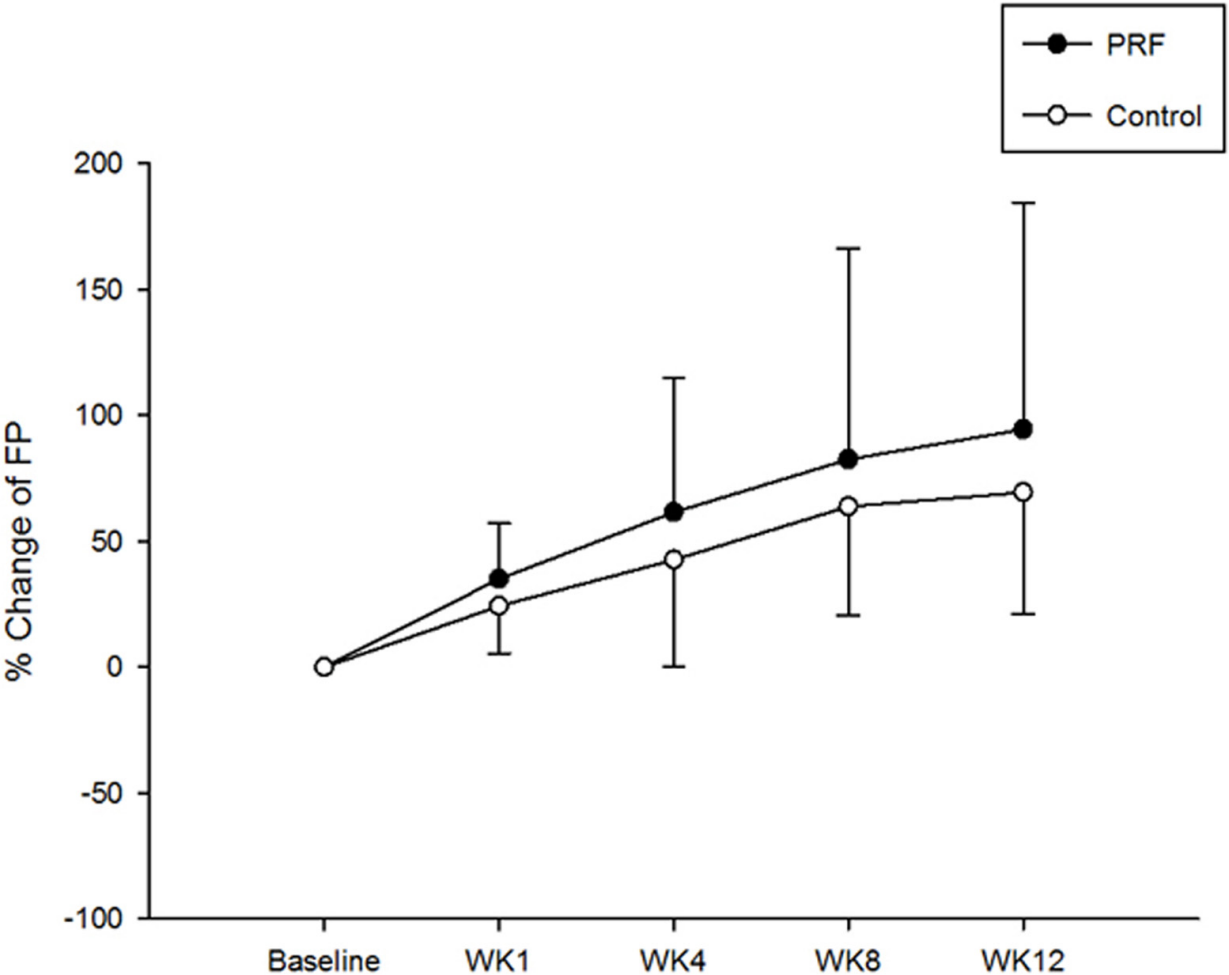
The percentage change in finger pinch strength (mean ± standard deviation) in both groups at different times.

Although a significant reduction of pain was revealed in both groups, decrease of the VAS score was higher at each follow-up period in the intervention group (Fig 4; mean ± standard deviation, -55.7 ± 14.9%, -61.6 ± 18.5%, -67.5 ± 18.9%, -77.2 ± 15.1% at week 1, 4, 8, and 12, respectively, in PRF vs. -20.8 ± 19.1%, -29.8 ± 25.4%, -32.6 ± 27.6%, -36.7 ± 32.5% in control, all pairwise comparisons having p < 0.001). Similarly, an increased strength in finger pinch was observed in both groups at each follow-up period (Fig 5) (35.1 ± 22.2%, 61.6 ± 53.5%, 82.6 ± 83.7%, 94.5 ± 90.2% in PRF vs. 24.3 ± 18.8%, 42.7 ± 42.6%, 63.9 ± 43.4%, 69.5 ± 48.5%, in control, at week 1, 4, 8, and 12, respectively, p > 0.05). Therefore, an additional 36% decrease in pain severity (VAS score) and 19% increase of finger pinch strength were observed after the intervention treatment.

No serious side effects or complications were detected in the two groups except for mild tingling or pain at the puncture site of the PRF treatment in three patients whose symptoms disappeared spontaneously half an hour later. No patient received medicine or any other treatment during the course of study.

## Discussion

To our best knowledge, the present study is the first prospective, randomized, single-blinded, controlled study to investigate the benefits of UG PRF for treating CTS. Compared to the control group managed with night splint only, the UG PRF intervention group experienced a faster (median onset time of 2 days vs. 14 days; hazard ratio = 7.37) and more robust pain reduction at each follow-up until the 12^th^ week.

The use of PRF was first introduced for chronic pain relief in 1998 by Sluijter et al. [17], and subsequent studies verified that it was a safe, nondestructive, repeatable, and long-lasting treatment for various pain-associated conditions [6–8]. Although the mechanism of PRF is not yet discovered, it has been suggested that PRF may modify neuronal membranes [6]. Moreover, PRF selectively affects the small diameter C- and A-δ fibers [18] and increased c-Fos immunoreactivity for 1 week after treatment in rat cervical dorsal root ganglia [19]. However, studies describing the applications of PRF for the treatment of CTS are scarce, and only one case report has been published so far [9]. Our results show that ultrasound-guided PRF leads to a more rapid and effective pain alleviation and functional recovery in patients with CTS.

CTS is caused by elevated pressure in the carpal tunnel that gradually leads to ischemia and mechanical deformation of the median nerve [2]. CTS is treated using conservative strategies, such as splinting, medication, steroid injection, and physical therapy, and with surgical decompression of the median nerve. Among these, splinting is the most popular method that fixes the wrist in an adequate position to lessen the pressure and compression in the median nerve [4].

Ultrasound guidance is gaining attention recently as an alternative to regional anesthesia. UG injections are a better solution compared to regional nerve blocks because the direct visualization of nerves allows direct stimulation of the target nerves, i.e. median nerve in this study. Therefore, onset and duration of nerve blocks is enhanced and the possible neuronal trauma is minimal [20]. In addition, patients and staff are at no risk of radiation, and ultrasound is less expensive and more convenient than Computed Tomography or fluoroscopy, which are traditionally used for nerve block. A study reported that UG steroid injections contributed to a more rapid and immense relief in CTS symptoms than blind injections [21]. The noticeable diminishment of VAS scores was observed soon after the UG PRF treatment in all subjects. Although UG is beneficial, the major disadvantage of UG injections is user dependency.

Ultrasound has been introduced into CTS diagnosis in recent years, and it is the most widely applied method for the accurate measurement of CSA at the pisiform level within the carpal tunnel [10, 22]. El Miedany et al. [22] recommended the use of CSA for diagnosing mild (10.0–13.0 mm^2^), moderate (13.0–15.0 mm^2^), and severe (> 15.0 mm^2^) forms of CTS. Most studies established a relationship between CSA of the median nerve, BCTQ scores, and hand function [22, 23]. On the other hand, some studies have reported no association between the symptom severity or functional status score and CSA of the median nerve [24, 25]. In our study, we found that the diminished CSA of the median nerve was accompanied with an improvement of VAS and BCTQ scores and finger pinch strength in both groups.

Studies have reported mixed results about correlations between electrophysiological tests and symptom severity or functional status scores in patients with CTS. Some authors suggested a close relationship [26, 27], while others proposed the opposite [28]. However, these variations are expected considering that routine electrophysiological tests mainly examine large myelinated fibers rather than small sensory fibers that may be responsible for some symptoms of CTS [29]. In our study, change in SNCV of the median nerve does not support this relationship, although significant improvement of VAS and BCTQ scores, CSA of the median nerve, and finger pinch strength was observed in both groups.

### Study Limitations

There are some limitations to this study. First, the number of cases is relatively small to make a clear conclusion. Second, due to the invasive nature of PRF, sham-controlled treatment procedures were unfeasible and unacceptable. However, treatment bias can hardly explain the significant effect of PRF that lasted until the 12^th^ week. Therefore, further studies are warranted to verify the current results. Moreover, SNCV of the median nerve alone does not provide sufficient details for an electrophysiological study. Finally, we did not investigate the possible long-term improvement of VAS and BCTQ scores beyond the 12^th^ week. Nevertheless, the significant difference in VAS scores between the two groups until the 12^th^ week (Table 2 and Fig 4) imply that the therapeutic effect of PRF may extend beyond the follow-up period examined in this study.

### Conclusions

Our results show that ultrasound-guided PRF is an effective, practical, and fast method to relieve pain in patients with CTS. This simple and reproducible procedure could be a potentially novel approach for treating CTS. However, further prospective clinical trials are encouraged with larger subject populations, sham-controlled treatment, and longer follow-up periods.

## Supporting Information

S1 CONSORT Checklist CONSORT Checklist. (DOC)

S1 Appendix Boston Carpal Tunnel Syndrome Questionnaire (BCTQ). (PDF)

S1 Protocol Trial protocol. (PDF)

S2 Protocol Trial protocol (Chinese version). (PDF)
