# Supplementary material for: Ultrasound-Guided Pulsed Radiofrequency for Carpal Tunnel Syndrome: A Single-Blinded Randomized Controlled Study
Source: PLoS One. 2015 Jun 12;10(6):e0129918. doi: 10.1371/journal.pone.0129918 (PMC4466776; doi:10.1371/journal.pone.0129918)
Supplement: S1 Appendix — (PDF) [file pone.0129918.s002.pdf]

Boston Carpal Tunnel Syndrome Questionnaire (BCTQ)

(一) Symptom severity scale (11 items)

	1	2	3	4	5
1. How severe is the hand or wrist pain that you have at night?	Normal	Slight	Medium	Severe	Very serious
2. How often did hand or wrist pain wake you up during a typical night in the past two weeks?	Normal	Once	2 to 3 times	4 to 5 times	More than 5 times
3. Do you typically have pain in your hand or wrist during the daytime?	No pain	Slight	Medium	Severe	Very serious
4. How often do you have hand or wrist pain during daytime?	Normal	1-2 times / day	3-5 times / day	More than 5 times	Continued
5. How long on average does an episode of pain last during the daytime?	Normal	＜10minutes	10~60 Continued	＞60minutes	Continued
6. Do you have numbness (loss of sensation) in your hand?	Normal	Slight	Medium	Severe	Very serious
7. Do you have weakness in your hand or wrist?	Normal	Slight	Medium	Severe	Very serious
8. Do you have tingling sensations in your hand?	Normal	Slight	Medium	Severe	Very serious
9. How severe is numbness (loss of sensation) or tingling at night?	Normal	Slight	Medium	Severe	Very serious
10. How often did hand numbness or tingling wake you up during a typical night during the past two weeks?	Normal	Once	2 to 3 times	4 to 5 times	More than 5 times
11. Do you have difficulty with the grasping and use of small objects such as keys or pens?	Without difficulty	Little difficulty	Moderately difficulty	Very difficulty	Very difficult

(二) Functional status scale (8 items)：

	No difficulty	Little difficulty	Moderate difficulty	Intense difficulty	Cannot perform the activity at all due to hands and wrists symptoms
Writing	1	2	3	4	5
Buttoning of clothes	1	2	3	4	5
Holding a book while reading	1	2	3	4	5
Gripping of a telephone handle	1	2	3	4	5
Opening of jars	1	2	3	4	5
Household chores	1	2	3	4	5
Carrying of grocery basket	1	2	3	4	5
Bathing and dressing	1	2	3	4	5
