# Supplementary material for: Ultrasound-Guided Pulsed Radiofrequency for Carpal Tunnel Syndrome: A Single-Blinded Randomized Controlled Study
Source: PLoS One. 2015 Jun 12;10(6):e0129918. doi: 10.1371/journal.pone.0129918 (PMC4466776; doi:10.1371/journal.pone.0129918)
Supplement: S1 Protocol — (PDF) [file pone.0129918.s003.pdf]

Study protocol

Title: Efficacy of Pulsed Radiofrequency of the Median Nerve under Ultrasound Guidance in Patients with Carpal Tunnel Syndrome

Background and Purpose:

Carpal tunnel syndrome (CTS) is the most common peripheral entrapment neuropathy. Although many conservative forms of management including the use of wrist splint, steroid injections and therapeutic ultrasound are applicable, their effectiveness is typically insignificant or short-lived.

Pulsed radiofrequency (PRF) treatment, a relative novel pain intervention at recent decade, was found to be able to alleviate pain for certain kinds of chronic pain conditions without damaging nerve. Ultrasound has been increasing used and attracting attention as an alternative to regional anesthesia in recent years. UG injection offer improved the quality and accuracy of regional nerve blocks because direct visualization of nerves allow for a direct stimulation of the target nerves. However, the application of PRF in CTS is scarce. The purpose of this study was to assess the analgesic effect and prognosis of ultrasound-guided PRF in the median nerve in patients with CTS.

Study design

Duration: 2012/1/1 to 2012/12/31.

Subjects:

Outpatient subjects who had typical symptoms and signs of CTS, such as positive Tinel's sign or Phalen's test and numbness/tingling in at least two of the first, second, and third digits and were all confirmed by electrophysiological study, were considered and enrolled. The patients who had conditions mimicking CTS, such as cervical radiculopathy, polyneuropathy, brachial plexopathy, thoracic outlet syndrome or who had previous wrist surgery or steroid injection for CTS, were all excluded.

Patient number: 40.

Methods:

The enrolled patients were randomized into two groups as the intervention group, who received one dose of PRF and the control group, who did not received RPF treatment by using drawing sealed envelope. To provide fundamental medical care of CTS, a wrist night splint was prescribed for each subject in both groups. Patients were ordered to wear the splint while resting

at night and at least 8 hours per day during the period of study. All procedures were conducted by single physician. All the measurements were performed by single physician who was blinded for the randomization, and the evaluation time was performed before intervention as well as the 1st, 4th, 8th and 12th weeks after treatment. All patients were instructed to keep away from getting any other treatments for their pain or discomfort resulting from CTS including analgesic agents, injection or acupuncture etc. during the period of follow-up. They were asked to notify us if they had taken these therapies.

PRF intervention:

Musculoskeletal ultrasonography (Terason, t3000, USA) was performed by the same physician. Patients were seated in a relaxed position with their forearm and finger resting on the table. The palm facing up and the median nerve were identified at the line of the proximal carpal tunnel (pisiform level). A 54-mm radiofrequency probe with a 4-mm active tip (Neurotherm NT1000, Neurotherm Inc., USA) was then advanced with UG using in-plane technique toward the median nerve. Sensory and motor stimulation were tested when the needle was close to the median nerve. Sensory stimulation (50-100 Hz; 1 ms pulsed width; up to 0.5 volt) was tested and the patients reported paresthesia in

the distal fingers. After performing motor stimulation (2 Hz; 1 ms pulsed width; up to 1 volt), the contractions of the thenar muscle were observed. PRF lesioning was then carried out for 120 seconds at a 2 Hz frequency and pulse width of 20 ms at 42℃. A total of 30 minutes of observation was conducted after the PRF lesioning procedure. The patients were discharged with no significant complications such as pain or bleeding.

Outcome measurements:

1. **Visual analog scale (VAS):** The severity of digital pain during any activity most time per day was marked down in the pain scale. Each patient reported the VAS score every day at the same time after the initial treatment until 2 consecutive days reaching the definition of onset time which was defined as the day the VAS score had declined by 40% or more.
2. **Boston Carpal Tunnel Syndrome Questionnaire (BCTQ).**
3. **Cross-sectional area of the median nerve:** It was measured at the proximal inlet of the carpal tunnel (level with the pisiform bone) by the same physician.
4. **Sensory nerve conduction velocity (SNCV) of median nerve.**
5. **Finger pinch**.

Data analysis:

Demographic data were analyzed by the Mann-Whitney U test for continuous data and X^2 test for categorical data. Wilcoxon's signed rank test was used to compare the outcome measures within each group of patients. The outcomes at each follow-up period were compared with the baseline values and differences between both groups were investigated using the Mann-Whitney U test. Comparability of change of VAS score and finger pinch measurements among the two groups was assessed using Mann-Whitney U test. Statistical significance was set at $p < 0.05$.

計畫聯絡人資料	
聯絡人姓名：陳 良 城（Liang-Cheng Chen）	
服務機關/公司行號/受託研究機構 (CRO)：<br>三軍總醫院復健部	職稱：三軍總醫院復健部職能治療科主任
聯絡電話：02-87923311 分機 12814<br>手機：0968395347　傳真：02-27936049	電子郵件：clctsgh@yahoo.com.tw
地址：台北市內湖區成功路 2 段 325 號	
主持人簽章/日期	
