# Supplementary material for: Ultrasound-Guided Pulsed Radiofrequency for Carpal Tunnel Syndrome: A Single-Blinded Randomized Controlled Study
Source: PLoS One. 2015 Jun 12;10(6):e0129918. doi: 10.1371/journal.pone.0129918 (PMC4466776; doi:10.1371/journal.pone.0129918)
Supplement: S2 Protocol — (PDF) [file pone.0129918.s004.pdf]

三軍總醫院人體試驗審議會
人體試驗計畫申請書

一、計畫名稱：

腕隧道症候群患者接受腕部正中神經脈衝射頻電燒療法效益之評估

二、背景及研究目的

腕隧道症候群（carpal tunnel syndrome）是最常見的周邊神經病變，係正中神經(median nerve)在腕隧道中因神經病變而引發手部的不適，除了影響日常生活功能及造成職場上的失能，也耗費非常多的醫療資源。發生率在男性隨著年齡增加而上升，而女性好發年齡在 45 到 54 歲之間[1]。有研究指出，男性盛行率約在 1%，而女性較高，為 7%[2]。症狀包括正中神經支配手部區域三指中（拇指、食指、和中指），至少有二指感覺到麻木（numbness）、刺痛（tingling）、灼熱（burning）、或疼痛不適等[3]。可經由臨床症狀、理學檢查、及神經電生理檢查來確認診斷[4]。雖然大部分病例發生原因不明，然而有許多因素可能導致疾病發生，其中包括腕隧道內病灶、感染、神經病變、和先天疾病等[5]。另外有研究指出，反覆性的壓迫、肥胖、節食、行子宮切除術後、停經、及矮小身材等，皆是腕隧道症候群的危險因子[6]。

治療方法包括保守性及外科手術治療。諸如藥物、腕部支架(splinting)、

超音波熱療、及復健等均屬於保守性治療項目[7]。在美國境內，每年均有 50 萬例接受外科手術治療，成功率有 70%~90%[8]。接受手術治療復發率約 19%，而再次手術治療的比例為 12%，原因多為手術不確實、纖維化增生、或是復發性腱鞘炎(recurrent tenosynovitis)[9]。

脈衝射頻電燒療法（pulsed mode radiofrequency，PRF）是以高頻熱凝之方式，阻斷痛覺神經傳導。美國及歐洲等國家，在十九世紀中期即不斷嘗試以高頻熱凝方式，利用絕緣套管配合電燒灼針，以 X-光透視觀察在反應痛覺之神經位置上，用八十度燒灼二至三分鐘止痛。到二十世紀初，更發展出新技術，以四十二度脈衝之方式，刺激神經節位置，更廣泛應用在下背痛，脊椎手術後遺症候群，上肢及下肢酸麻痛…等，效期可維持六至十二個月。以四十二度之脈衝方式治療，不致燒損神經，可安全而立即有效緩解疼痛不適，而且療效可持續一段很長時間[10-12]。此療法的機轉，目前仍有爭議，尚未定論。學者研究指出，在老鼠頸部背根神經節，經過脈衝射頻電燒治療後，發現 C-Fos 免疫反應增強，而且可持續一周之久[13]。不論是實驗室內或是活體研究，脈衝射頻電燒療法均不會對局部神經產生破壞作用。Erdine 等人研究顯示，射頻電燒療法有生物效應(biological effect)，不會產生明顯的熱能破壞，而且可以直接作用在 C& δ 痛覺纖維上[14]。

以超音波定位施以局部麻醉漸被廣泛付用，且獲得良好的神經阻斷效果。直接可見欲治療的神經影像，可以避免神經受到傷害，並且可以加速神經阻斷的起始時

間(onset time)及延長有效治療的時期[15]。近幾年來，針對慢性疼痛患者，在超音波定位下施以介入性治療(intervention)，已逐漸受到重視。然而在超音波定位下，直接看到周邊神經、並施以脈衝射頻電燒療法卻仍未見到相關研究。

本研究為第一個利用即時且高解析度的超音波導引下，施以腕隧道內正中神經脈衝射頻電燒治療，期能提供臨床上更安全且快速及更有效的治療方式，嘉惠更多患者。

三、研究方法

1. 執行期間：自 101 年 1 月 1 日起至 101 年 12 月 31 日止。
2. 研究對象：門診中收取 40 位 18 歲以上罹患腕隧道症候群患者，症狀持續三個月以上，且排除頸神經根病變及開刀病史。
3. 病人隨機分配分成 2 組，第一組只接受副木固定，持續 3 個月時間，此組為對照組。另一組則先接受一次腕部正中神經脈衝式射頻電燒療法後，再接受如同上述的副木固定 3 個月。兩組患者在治療前均先經過檢查評估，進行參數測量。治療後第一週、一個月、二個月及三個月回診追蹤，並由同一位醫師負責評估(該醫師並不知道分組結果)。追蹤期間病患不得接受其他治療方式，包括藥物、注射、針灸及手術等，萬一有接受其他治療務必告知研究醫師。兩組患者在治療前均先經過檢查評估，進行參數測量。
4. 超音波擺位:

患者採坐姿，以右手治療為例，操作者坐在患者對面，機器置於右前方，先將鉤骨的鉤突極大角突出用筆標示出來，接著皮膚無菌消毒。超音波探頭［HFL 13-6 MHz，38 mm broad band linear array（Sonosite Micromaxx lnc., Bothell,WA）］套上無菌套頭，使用無菌凝膠作為介質。患者手腕伸直，檢查者以觸摸先診尺骨側突出鉤骨的鉤突(hook of hamate)，於橈角側摸出大角骨(trapezium)的突出，將探頭置放其上即可掃描出腕隧道，若不清楚可將探頭往近端平移。正中神經呈現中低回音的構造，內含高回音的點狀物。

5. 射頻電燒止痛儀操作:

使用射頻電燒止痛儀（Neurotherm NT1000, Neurotherm Inc., USA），將 21 號 5 公分長的電燒探針，順著超音波束的縱軸方向由斜方插入，在超音波導引下，將針尖放置腕隧道內正中神經處。先開啟主機 “stimulation sensor” 感覺神經測試，設定 0.5 volt，50-100 Hz 功率，此時病人會感覺到手部痠麻。再開啟主機“stimulation motor”運動神經測試，設定 1 volt，2Hz 功率，觀察到拇指魚際肌（thenar muscle)的肌肉收縮即是接近神經位置。定位完成後條件設定改為 45 voltage，開始 90 秒（180 次輸出）的電刺激。開始時針尖溫度為 38 ℃，溫度會慢慢上升，在 30 秒內溫度可上升至 42 ℃。術後 30 分鐘，進行各項參數測量。在確認沒有流血併發症後，始完成整個程序。

6. 評估及統計方法

a. 主要評估項目包括 visual analog scale（VAS），分數由 0（無痛）至 10 分

（極痛），當 VAS 下降≧40%時，代表疼痛有顯著的改善。

b. 腕部失能指數採用 Boston Carpal Tunnel Syndrome Questionaire（BCTQ）[16]。

c. 超音波正中神經截面積[17]。

d. 正中神經傳導。

e. 手指捏力評估。

f. 以 Mann-Whitney U test and r 及 X^2 test 做為統計分析，比較兩組病患基本資料；Wilcoxon’s signed rank test 統計治療後各項數值與 baseline 進步情形；Mann-Whitney U test 比較實驗組各項進步情形是否優於對照組，*p* 值小於 0.05 代表統計學上有顯著意義。

四、可能傷害及處理

無明顯副作用或可能傷害發生。若扎針處有疼痛及出血，則可用冰敷 10 至 20 分鐘來緩解不適症狀。

五、參考文獻

1. Stevens JC, Witt, JC, Smith BE, Weaver AL. Carpal tunnel syndrome in Rochester, Minnesota, 1961 to 1980. Neurology 1988;38:134-138.
2. Atroshi S, Gummesson C, Johnson R, Ornstein E, Ranstam J, Rosen I. Prevalence of carpal tunnel syndrome in a general population. JAMA 1999; 282:151-158.

3. Rempel D, Evanoff B, Amadio P C, de Krom M, Franklin G, Franzblau A, et al. Consensus criteria for the classification of carpal tunnel syndrome in epidemiologic studies. Am J Public Health 1998;88:1447-1451.
4. Practice parameter for electrodiagnostic studies in carpal tunnel syndrome: Summary statement. Muscle Nerve 2002;25:918-922.
5. von Schroeder HP, Botte MJ. Carpal tunnel syndrome. Hand Clin 1996;12: 643-655.
6. de Krom MC, Kester AD, Knipschild PG, Spaans F. Risk factors for carpal tunnel syndrome. Am J Epidemiol 1990;132:1102-1110.
7. Muller M, Tsui D, Schnurr R, Biddulph Deisroth L, Hard J, MacDermid JC. Effectiveness of hand therapy interventions in primary management of carpal tunnel syndrome: A systematic review. J Hand Ther 2004;17:210-228.
8. Jerosch-Herold C, Leite JC, Song F. A systematic review of outcomes assessed in randomized controlled trials of surgical interventions for carpal tunnel syndrome using the International Classification of Functioning, Disability and Health (ICF) as a reference tool. BMC Musculoskelet Disord 2006;7:96.
9. Botte MJ, von Schroeder HP Abrams RA, Geliman H. Recurrent carpal tunnel syndrome. Hand Clin 1996;12:731-743.
10. Chua NH, Vissers KC, Sluijter ME. (2011) Pulsed radiofrequency treatment in interventional pain management: mechanisms and potential indications-a review. Acta Neurochir (Wien) 153:

763-771.

11. Lakemeier S, Lind M, Schultz W, Fuchs-Winkelmann S, Timmesfeld N, et al. (2013) A comparison of intraarticular lumbar facet joint steroid injections and lumbar facet joint radiofrequency denervation in the treatment of low back pain: a randomized, controlled, double-blind trial. Anesth Analg 117: 228-235.
12. Gofeld M, Restrepo-Garces CE, Theodore BR, Faclier G. (2013) Pulsed radiofrequency of suprascapular nerve for chronic shoulder pain: a randomized double-blind active placebo-controlled study. Pain Pract 13: 96-103.
13. Van Zundert J, de Louw AJ, Joosten EA, Kessels AG, Honig W, et al. (2005) Pulsed and continuous radiofrequency current adjacent to the cervical dorsal root ganglion of the rat induces late cellular activity in the dorsal horn. Anesthesiology 102: 125-131.
14. Erdine S, Bilir A, Cosman ER, Cosman ER, Jr. (2009) Ultrastructural changes in axons following exposure to pulsed radiofrequency fields. Pain Pract 9: 407-417.
15. Sites BD, Brull R. (2006) Ultrasound guidance in peripheral regional anesthesia: philosophy, evidence-based medicine, and techniques. Curr Opin Anaesthesiol 19: 630-639.
16. Sambandam SN, Priyanka P, GulA, llango B. Critical analysis of outcome measures used in the assessment of carpal tunnel syndrome. mt Orthop 2007; 17 (EPub).
17. Wong SM, Griffith JF, Hui AC, Tang A, Wong KS. Discriminatory sonographic criteria for the diagnosis of carpal tunnel syndrome. Arthritis Rheum 2002;46:1914-1921.

計畫聯絡人資料	
聯絡人姓名：陳 良 城 (Liang-Cheng Chen)	
服務機關/公司行號/受託研究機構 (CRO)：<br>三軍總醫院復健部	職稱：三軍總醫院復健部職能治療科主任
聯絡電話：02-87923311 分機 12814<br>手機：0968395347　　傳真：02-27936049	電子郵件：clctsgh@yahoo.com.tw
地址：台北市內湖區成功路 2 段 325 號	
主持人簽章/日期	
